# Editorial: Microbiologically influenced corrosion (MIC): its mechanisms, technological, economic, and environmental impacts

**DOI:** 10.3389/fmicb.2023.1249565

**Published:** 2023-11-13

**Authors:** Reza Javaherdashti

**Affiliations:** Eninco Engineering B.V., Hengelo, Netherlands

**Keywords:** microbiologically influenced corrosion (MIC), microbiologically influenced deterioration (MID), sulfate-reducing bacteria (SRB), extracellular electron transfer (EET), corrosion, bacteria, archaea

Over the decades, MIC and its understanding have come a long way, yet many knowledge gaps remain. For instance, while we relatively know a lot about MIC for materials in water (particularly sea water), little is known about mechanisms by which MIC occurs in soil or even atmospheric- aided MIC (Parra et al., 1996; Maruthamuthu et al., 2008). While we have a good basic understanding of microbiologically influenced corrosion, we relatively know less about microbiologically influenced deterioration (MID), the former specific to metals and the latter to be used to address it for non-metals. On the hand, MIC studies used to be mainly revolving around the impact of sulfate-reducing bacteria (SRB), and have now moved to consider the importance of other groups of bacteria and archaea. An example is Pseudomonas family whose corrosive impact on engineering materials is known at least from 1962 (Eagon, 1962) and have been isolated from many oil and gas sites around the world (Bairi et al., 2023) their impact on accelerating corrosion via rapid biofilm (Temenos) formation (Mansouri et al., 2014; Javaherdashti, 2017).

Also, instead of putting emphasis on single type cultures of bacteria, now mixed cultures and their effects on corrosion are being studied. Before mid-1980s, the corrosive action of SRB was being explained by CMIC (Chemical microbiologically influenced corrosion) parameters, that is contribution of chemical species to this corrosion phenomenon. After that (and for the first time, it was discovered for SRB) it was found out that there could be another way of explaining the corrosive action of SRB in addition to many other theories through EMIC (Electrical Microbiologically influenced corrosion). EMIC, in simple terms, puts forward that bacteria attach themselves directly on the metal surface and take electrons directly from the metal via three mechanisms collectively referred to as Extracellular Electron Transfer (EET) (Reguera et al., 2005; Kato, 2016). Literally speaking, this way bacteria that receives electrons can be regarded as the cathode and the metal remains the anode.

A frequently used scale to measure the severity and intensity of corrosion is to measure the speed at which corrosion can progress (i.e., corrosion rate). Corrosion rate can be expressed in various units such as mpy (miles per year), ipy (inch per year) or mm/y (millimeter per year). While CMIC can explain observed corrosion rates of 16 mpy, EMIC can provide mechanistic evidence for cases where the observed corrosion rate is 36 mpy (Enning and Garrelfs, 2014). EMIC has been observed bacteria other than SRB such Nitrate-reducing bacteria (Rajala, 2017) and Methanogens (Kato, 2016).

In this Research Topic collection of papers, the contributing researchers have looked at the problem of MIC from different aspects and in very innovative ways. In many MIC studies, the tradition has it that the studied material has been carbon steel and the bacteria will pure or mixed cultures of SRB. Two of the research papers do consider MIC in steel, but in a different context: the paper by Mihaela Stancu on MIC of carbon steel by Stenotrophomonas and Bacillus is an attempt to study the impact of non-SRB on carbon steel and is expected to spark discussions among MIC professionals. The paper by Shi et al., also considers MIC of X65 steel. Researchers who are mainly interested in studying the performance of ferrous materials against microbial species, will find those papers interesting and significant. Undoubtedly, one of the most believed issues in industry is legendary resistance of copper and its alloys against MIC. This that stems from the fact that copper, along some other metals such as silver and molybdenum are known to have toxic effects on microorganisms (Kato, 2016). The review by Amendola and Acharjee on MIC of Copper and its alloys and also the paper by Li et al. on MIC of Cupro-nickel alloys are two very important papers in this field. The latter along with the paper by Yang et al. can also be assessed among pioneering works on the role of Pseudomonas bacteria on MIC, in this regard these papers are expected to take the attention of professionals to MIC-related bacteria other than SRB.

Due to the very nature of MIC which is reflected in both industry and research activities, our aim has been to have an equilibrium between academic research interests and industrial applications., thus this RT is pleased to include several contributions from industrial researchers. Papers by Shi et al. (off-shore industry) and Stamps et al. (Biodeterioration in Biodiesel storage tanks) can be mentioned. The latter paper is an investigation of symbiotic action of fungal and bacterial proliferation in inducing MIC. This is another example novel interactions that can stretch the existing boundaries of MIC research. Another new avenue of research on MIC is modification of surfaces that are susceptible to MIC, and nanotechnology has proved to offer new innovations, in this regard. This technology can also be used in chemical treatment of MIC.

While this Research Topic offers many new insights into MIC, there are still many aspects of MIC that I would still hope to see more of. Topics such as the economy and ecology consequences of ignoring MIC/MID, or more detailed discussions on MIC/MID mechanisms, advanced modeling of MIC/MID and many more. I hope that in the future these topics and many more will be covered.

I would like to sincerely thank Mr. Colin N Shaw, Research Integrity Specialist at Frontiers, for his invaluable support and comments. Also, my sincere thanks go our topic editors Prof. Dr. Judit Telegdi (Hungary) and Prof. Dr. Raman Singh (Australia) for their supports and kind contributions, in addition to our reviewers who assisted us to select the best possible papers. Our highest thanks and appreciations go to our authors who kindly shared their experiences with us and with the readers of this Research Topic. My highest personal appreciation goes to past and present individuals who at Frontiers assisted in bringing this Research Topic from an idea to a reality: Commissioning Specialists, particularly Ms. Katrin Ansorge and her tireless efforts to facilitate and ease-off the procedure for us. It goes without saying that if it was not for Frontiers support, this publication would never come to life; I am very glad that Frontiers believed in me and allowed me to use their resources to make such a unique contribution to MIC industry.

## Author contributions

The author confirms being the sole contributor of this work and has approved it for publication.
